# Dynamic stimuli demonstrate a categorical representation of facial expression in the amygdala

**DOI:** 10.1016/j.neuropsychologia.2014.01.005

**Published:** 2014-04

**Authors:** Richard J. Harris, Andrew W. Young, Timothy J. Andrews

**Affiliations:** Department of Psychology and York Neuroimaging Centre, University of York, York YO10 5DD, United Kingdom

**Keywords:** Face, Expression, Emotion, fMRI

## Abstract

Face-selective regions in the amygdala and posterior superior temporal sulcus (pSTS) are strongly implicated in the processing of transient facial signals, such as expression. Here, we measured neural responses in participants while they viewed dynamic changes in facial expression. Our aim was to explore how facial expression is represented in different face-selective regions. Short movies were generated by morphing between faces posing a neutral expression and a prototypical expression of a basic emotion (either anger, disgust, fear, happiness or sadness). These dynamic stimuli were presented in block design in the following four stimulus conditions: (1) *same-expression change, same-identity*, (2) *same-expression change, different-identity*, (3) *different-expression change*, *same-identity*, *and* (4) *different-expression change*, *different-identity*. So, within a *same-expression change* condition the movies would show the same change in expression whereas in the *different-expression change* conditions each movie would have a different change in expression. Facial identity remained constant during each movie but in the different identity conditions the facial identity varied between each movie in a block. The amygdala, but not the posterior STS, demonstrated a greater response to blocks in which each movie morphed from neutral to a different emotion category compared to blocks in which each movie morphed to the same emotion category. Neural adaptation in the amygdala was not affected by changes in facial identity. These results are consistent with a role of the amygdala in category-based representation of facial expressions of emotion.

## Highlights

• How facial expression is represented remains controversial.
• Responses to facial expressions were measured with fMRI using dynamic stimuli.
• The amygdala shows a categorical representation of facial expression.

## 1 Introduction

Transient changes in facial musculature that signal current emotional state are critical for effective social interactions. A prominent model of face perception has proposed that a neural pathway from the occipital face area (OFA) to the posterior superior temporal sulcus (pSTS) is involved in processing transient facial signals such as facial expression and eye gaze. In this model the STS is thought to have reciprocal connections with the amygdala which is recruited for further analysis of facial expression (Haxby, Hoffman, & Gobbini, 2000). The sensitivity of the STS and amygdala to a range of facial expressions has been demonstrated across a variety of experiments (Adolphs, Tranel, Damasio, & Damasio, 1994; Adolphs et al., 1999; Andrews & Ewbank, 2004; Baseler, Harris, Young, & Andrews, 2013; Engell & Haxby, 2007; Harris, Young, & Andrews, 2012; Narumoto, Okada, Sadato, Fukui, & Yonekura, 2001).

>However, relatively little is known regarding how facial expression is encoded in these regions. Models of facial expression perception have debated whether facial expressions are represented as belonging to discrete categories of emotion or as gradations along continuous dimensions (see Bruce & Young, 2012). Although usually treated as incompatible opposites there is evidence for both accounts. Evidence for categorical perception of expression is shown by the consistency with which basic emotions are recognized (Ekman, 1972) and by the increased sensitivity to changes in facial expression that alter the perceived emotion (Calder, Young, Perrett, Etcoff, & Rowland, 1996; Etcoff & Magee, 1992). In contrast, continuous or dimensional models are better able to explain the systematic confusions that occur when labeling facial expressions (Woodworth & Schlosberg, 1954). Continuous models can also account for the fact that we are readily able to perceive differences in intensity of a given emotional expression (Calder, Young, Rowland, & Perrett, 1997; Young et al., 1997) and for variation in the way that basic emotions are expressed (Rozin, Lowery, & Ebert, 1994).

Previously, we offered evidence supporting a synthesis of the above accounts at the neural level by demonstrating that expression is represented in the brain in both a categorical and a continuous manner (Harris et al., 2012). Specifically, by morphing static images of faces to create equal physical changes between images that either fell within the same emotion category or crossed the boundary between different emotion categories, we showed that the amygdala is more sensitive to between than within-category changes (showing a more categorical representation of facial expression) whereas the pSTS is equally sensitive to within and between-category change (indicating a more continuous representation).

In this current study, we aimed to further explore the categorical representation of expression in the amygdala using dynamic stimuli. Dynamic changes in facial expression can provide a stringent test of categorical representations, as dynamic movies necessarily incorporate continuous transient changes in expression, and many of these changes need to be disregarded in order to assign dynamic expressions into discrete categories. We used short movies that always showed a change from a neutral resting expression to an intense emotional expression. These movies were created by animating morphed images of facial expressions of basic emotions from the Ekman and Friesen (1976) series. We then used a block fMR-adaptation design to compare neural responses to blocks involving a series of these short movies in which the final expressions were either the same (e.g. all fear) or different (mixed emotions). So within a block participants we saw a series of movies which displayed a dynamic change from a neutral expression to the apex of an emotion. In the same-expression change conditions the same change in expression was displayed across all movies (e.g. all neutral to fear). In the different expression conditions each movie had a different facial expression change (neutral to fear, neutral to disgust, neutral to happy etc). These same and different expression blocks could be presented with either the same or different facial identity. In the same identity conditions each movie would show the same person across the block, whilst in the different identity conditions each movie would show a different person.

This design incorporates contrasts that provide substantial criteria for a category-based response to moving expressions. A neural region using a predominately categorical representation of expression should show a greater response to the different compared to the same change in expression conditions, as these conditions involve a change in the emotion category. Moreover, a region showing a response based primarily on emotional categories should also be relatively insensitive to changes in facial identity. However, if a region does not represent expression into emotion categories it should respond equally to the same and different expression blocks, because all of the movies are based on morphed sequences of images that undergo continuous changes. From Harris et al.’s (2012) results with static expressions, we predicted that the amygdala, but not the pSTS, would demonstrate a categorical representation of expression.

## 2 Method

### 2.1 Subjects

Nineteen participants took part in this experiment (14 females; mean age, 23). All participants were right-handed and had normal or corrected-to-normal vision. Visual stimuli (8°×8°) were back-projected onto a screen located inside the magnetic bore, 57 cm from subjects′ eyes. All subjects provided written informed consent and the study was given ethical approval by the York Neuroimaging Centre Ethics Committee.

### 2.2 Localiser scan

A functional localiser was used to independently identify regions of interest. This localiser involved a block design with five different conditions: (1) *faces*, (2) *bodies*, (3) *inanimate objects*, (4) *places*, and (5) *scrambled images* of the former categories. Face images were taken from the Psychological Image Collection at Stirling (PICS; http://pics.psych.stir.ac.uk/). These images varied in viewpoint (frontal, $\frac{3}{4}$ view, profile) and expression (neutral, happy, speaking) within a block. Both male and female faces were used. Body images were taken from a collection at the University of Bangor (http://www.bangor.ac.uk/~pss811/), and contained clothed male and female headless bodies in a variety of postures. Images of places consisted of a variety of unfamiliar indoor scenes, houses and buildings, city scenes and natural landscapes. Stimuli in the object condition consisted of different inanimate objects including tools, ornaments, and furniture. Fourier-scrambled images were created by randomizing the phase of each two-dimensional frequency component in the original image, while keeping the power of the components constant. Scrambled images were generated from the images used in the other stimulus categories.

Each stimulus block consisted of 10 images from a single stimulus condition. Each image within a block was presented for 700 ms and followed by a 200 ms blank screen, resulting in a total block length of 9 s. Stimulus blocks were separated by a 9 s gray screen with a central fixation cross. Each condition was repeated four times in a counterbalanced design resulting in a total scan length of 7.2 min. All participants viewed the same sequence of blocks and images. To ensure participants maintained attention throughout the experiment, participants had to detect the presence of a red dot superimposed onto 20% of the images. No significant differences in red dot detection were evident across experimental conditions (Accuracy: 96.5%, *F*_(1,18)_=0.71; RT: 673.4 ms, *F*_(1,18)_=1.95, *p*=0.18).

### 2.3 Experimental scan

#### 2.3.1 Stimuli

The initial face stimuli were Ekman faces selected from the Young et al. Facial Expressions of Emotion Stimuli and Tests (FEEST) set (Young, Perrett, Calder, Sprengelmeyer, & Ekman, 2002). Five individuals posing five expressions (anger, disgust, fear, happiness and sadness) were selected based on the following three main criteria: (i) a high recognition rate for all expressions (mean recognition rate in a six-alternative forced-choice experiment: 93% Young et al., 2002), (ii) consistency of the action units (muscle groups) across different individuals posing a particular expression, and (iii) visual similarity of the posed expression across individuals. Using these criteria to select the individuals from the FEEST set helped to minimize variations in how the expressions were posed.

The frames for the movies were generated by morphing between each individual′s neutral expression and each of their prototype expressions in 5% steps using PsychoMorph (Tiddeman, Burt, & Perrett, 2001). Movies were generated by playing the morphed images in sequence using Adobe Premiere Pro. The first (neutral) frame was played for 160 ms and the final frame (prototype expression) was played for 280 ms. The 18 intermediate frames were each played for 40 ms. Validation of the movie stimuli was demonstrated in an expression-classification experiment, in which recognition rates of the dynamic expressions were compared to the recognition rate for the equivalent original prototype expression. Participants either classified the static or dynamic expressions in a 5AFC task. 20 participants (11 females; mean age 29) rated the static expressions and 20 participants (12 females; mean age 27) rated the dynamic expressions. The static stimuli were shown for an equivalent amount of time as the dynamic stimuli and both were followed by a 2 s gray screen, during which participants could make their response. This experiment found that recognition accuracy for the static expression was 83.6% and for the dynamic expressions 84.3%.

#### 2.3.2 Procedure

The aim of this experiment was to investigate the nature of the representation of expression in the amygdala and pSTS. There were four conditions in this experiment which all involved blocks showing a sequence of movies each of which involved a dynamic change in expression from a neutral pose to a basic emotion: (1) *same-expression change, same-identity*, (2) *same-expression change, different-identity*, (3) *different-expression change, same-identity*, and (4) *different-expression change, different-identity*. The *same-expression change* conditions involved 5 movies all displaying the same change in expression (i.e. all neutral to the same emotion). In the *different-expression change* conditions each of the 5 movies displayed a change from neutral to a different basic emotion. Each movie was created using the face of a single model (identity). In the *same-identity* conditions the same identity was shown in each of the 5 movies, and in the *different-identity* conditions each of the 5 movies had a different facial identity. The movie stimuli were presented in blocks, with 5 movies per block. Each movie was presented for 1160 ms and separated by a gray screen presented for 200 ms. Successive stimulus blocks were separated by a 9 s fixation gray screen. Each condition was presented 10 times in a counterbalanced order, giving a total of 40 blocks. This resulted in total scan duration of 10.5 min. To ensure participants maintained attention throughout the experiment, participants had to push a button when they detected the presence of a red dot, which was superimposed onto 20% of the movies. No significant differences in red dot detection were evident across experimental conditions (Accuracy: 96.0%, *F*_(1,18)_=0.14; RT: 646.7 ms, *F*_(1,18)_=0.35).

### 2.4 Imaging parameters and fMRI analysis

Data was collected using a GE 3T HD Excite MRI scanner at York Neuroimaging Centre at the University of York. A Magnex head-dedicated gradient insert coil was used in conjunction with a birdcage, radio-frequency coil tuned to 127.4 MHz. A gradient-echo EPI sequence was used to collect data from 38 contiguous axial slices (TR=3, TE=25 ms, FOV=28×28 cm^2^, matrix size=128×128, slice thickness=4 mm). These were co-registered onto a T1-weighted anatomical image (1×1×1 mm^3^) from each participant. To improve registrations, an additional T1-weighted image was taken in the same plane as the EPI slices. Statistical analysis of the fMRI data was performed using FEAT (http://www.fmrib.ox.ac.uk/fsl). The initial 9 s of data from each scan were removed to minimize the effects of magnetic saturation. Motion correction was followed by spatial smoothing (Gaussian, FWHM 6 mm) and temporal high-pass filtering (cutoff, 0.01 Hz).

Face-selective regions were individually defined in each individual using the localiser scan by the average of the following contrasts: face>body, face>object, face>place and face>scrambled. Statistical images were thresholded at *p*<0.001 (uncorrected). In this way, contiguous clusters of voxels located in the inferior fusiform gyrus, in the posterior occipital cortex and in the superior temporal lobe of individual participants could be identified as the FFA, OFA and the pSTS respectively. A different approach had to be taken to define the amygdala. Signals in the anterior regions of the temporal lobe are typically noisy, because of the proximity to the ear canals. The lower within-participant signal-to-noise makes it difficult to determine face-selectivity in the amygdala at the level of individual participants. A face-responsive ROI in the amygdala was therefore defined from the face-selective statistical map at the group level, thresholded at *p*<0.001 (uncorrected). This ROI in the amygdala was then transformed into the individual MRI space for each participant. The time-course of response in the amygdala ROI was then evaluated for each participant to ensure that it responded more to faces than non-face stimuli. In all other respects, the data were processed in exactly the same way for all ROIs.

For the experimental scan, the time series of MR response from each voxel within a ROI was converted from units of image intensity to percentage signal change. All voxels in a given ROI were then averaged to give a single time series for each ROI in each participant. Individual stimulus blocks were normalized by subtracting every time point by the zero point for that stimulus block. The normalized data were then averaged to obtain the mean time course for each stimulus condition. The peak response was taken as an average of the TR 2 (6 s) and TR 3 (9 s) following the onset of each block.

## 3 Results

**Fig. 1:**
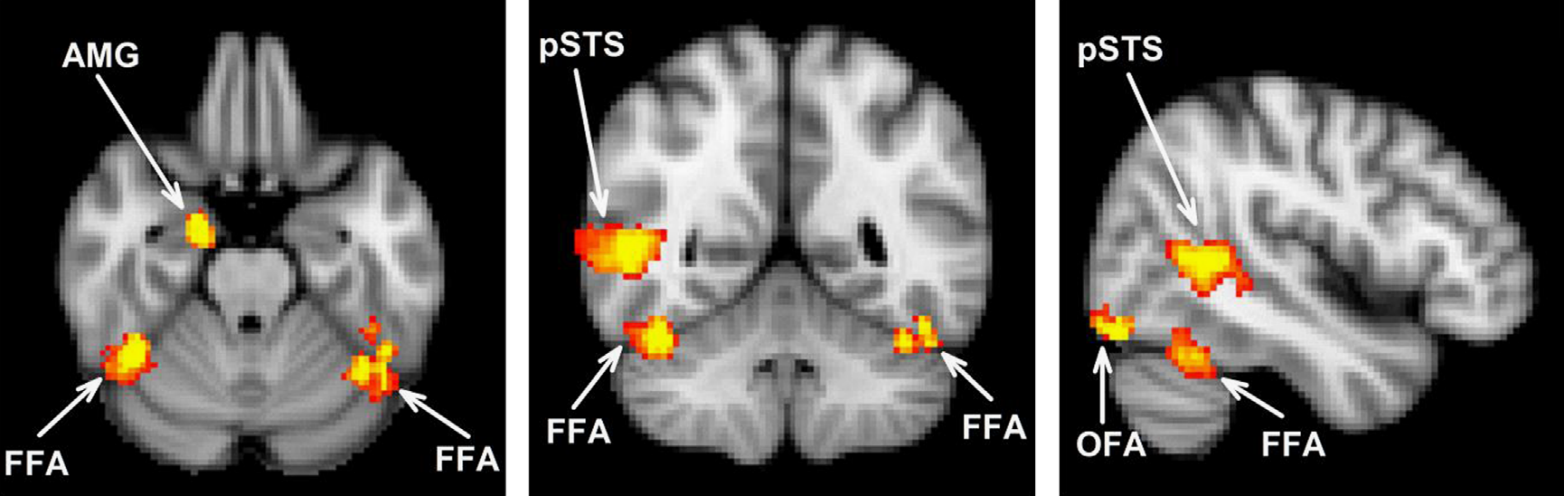
Location of regions that were more responsive to faces compared to non-face stimuli in the Localiser scan. MNI coordinates (mm) of slices: *x*=46, *y*=−52, *z*=−24. FFA: fusiform face area, OFA: occipital face area, pSTS: posterior superior temporal sulcus, and AMG: amygdala.

The location of all face-selective regions is shown in Fig. 1 and Table 1. The localiser was able to identify a posterior part of right STS and a region in the right amygdala which responded more to faces than to non-face stimuli. A further two regions, the OFA and FFA, also showed a preferential response to faces and were identified in both the left and right hemispheres. To determine whether there was any difference in the neural response across the hemispheres in the adaptation experiment, we conducted a 2×2×4 ANOVA with Hemisphere (left, right), Region (OFA, FFA) and Condition (same-expression change, same-identity, same-expression change, different-identity, different-expression change, same-identity, different-expression change, different-identity) as the main factors (participants in which the OFA and FFA could only be identified unilaterally (see Table 1), were not included in this ANOVA). There was no main effect of Hemisphere (*F*_(1,12)_=2.73, *p*=0.13). There was also no significant Hemisphere⁎Condition (*F*_(3,36)_=0.45), Hemisphere⁎Region (*F*_(1,12)_=0.25) or Hemisphere⁎Region⁎Condition (*F*_(3,36)_=1.17, *p*=0.34) interactions. Accordingly, for participants that demonstrated bilate ral OFA and FFA, the neural responses were combined across hemispheres.

Next, we determined whether there was any difference between the response in the face-selective regions to dynamic changes in facial expression and to changes in identity. A 4×2×2 ANOVA with Region (pSTS, amygdala, FFA, OFA) Expression (same, different) and Identity (same, different) as the main factors, revealed significant main effects of Expression (*F*_(1,14)_=7.30, *p*=0.02) and Region (*F*_(3,42)_=63.71, *p*<0.0001) and a marginal, but not statistically significant effect of Identity (*F*_(1,14)_=4.35, *p*=0.06). There was also a significant interaction between Region×Expression (*F*_(3,42)_=3.06, *p*=0.04).The main focus of the analysis is the pSTS and the amygdala as these regions have been previously implicated in the processing of facial expression (Harris et al., 2012; Haxby et al., 2000). To further investigate whether these two regions demonstrated a different pattern of response, we conducted a 2×2×2 ANOVA with the factors Region (amygdala, pSTS), Expression (same, different) and Identity (same, different). This revealed a significant main effect of Region (*F*_(1,14)_=48.42, *p*<0.001) and Expression (*F*_(1,14)_=5.67, *p*=0.03) but not identity (*F*_(1,14)_=0.01) There was also a significant Region⁎Expression interaction (*F*_(1,14)_=6.99, *p*=0.02), suggesting a dissociable representation of expression in these regions. Therefore, to investigate how the response to dynamic changes in facial expression differed between the face-selective regions, the patterns of response in the face-selective regions of interest were considered individually.

**Fig. 2:**
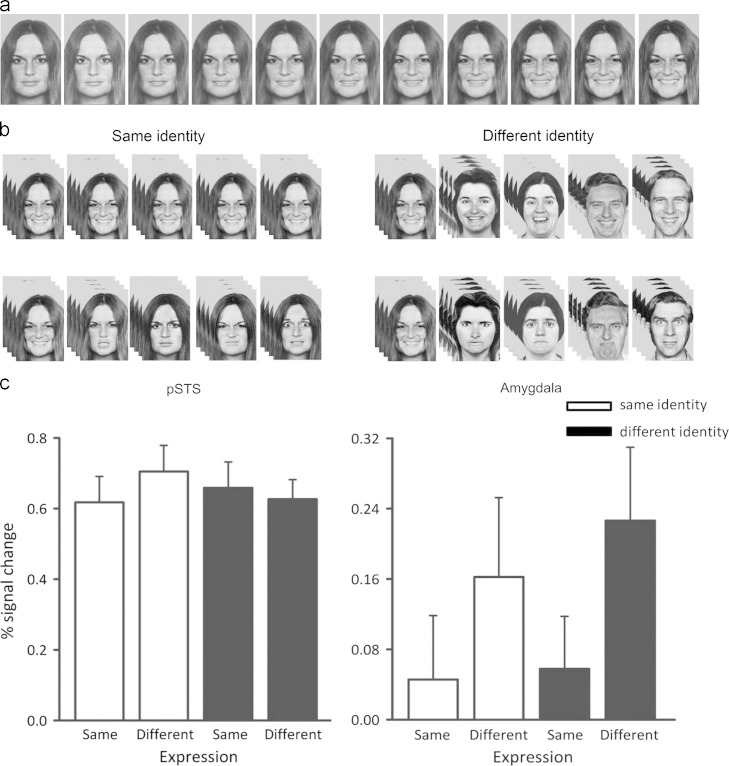
Example stimuli and neural responses to dynamic changes in facial expression: (a) alternate frames from an example neutral to happiness movie. (b) Example of the sequence of movies within a block from the four experimental conditions: (upper, left) same-expression, same-identity; (upper, right) same-expression, different-identity; (lower, left) different-expression, same-identity; (lower, right) different-expression, different-expression. (c) Peak responses to the different conditions in the pSTS and amygdala. The results show that the pSTS was responsive to all conditions, consistent with a continuous representation of facial expression. However, the amygdala was more sensitive to a series of expression movies which displayed different changes in the emotion category, demonstrating a more categorical representation of emotion.

Fig. 2c shows the peak responses in the posterior part of the right STS. A 2×2 ANOVA with the factors Expression (same, different) and Identity (same, different) revealed no significant effect of Expression (*F*_(1,17)_=0.66), or Identity (*F*_(1,17)_=0.20). There was also no significant Expression⁎Identity interaction (*F*_(1,17)_=1.97, *p*=0.18). In contrast, the amygdala was sensitive to blocks of faces in which the dynamic change in expression varied across the block. A 2×2 repeated measures ANOVA found a significant main effect of Expression (*F*_(1,15)_=5.10, *p*=0.04) but not Identity (*F*_(1,15)_=0.23). There was no significant interaction Expression⁎identity (*F*_(1,15)_=0.08). The main effect of Expression was due to the bigger response to the *different-expression* conditions compared to the *same-expression* conditions (different expression: 0.19%, same expression: 0.05%). This pattern held regardless of whether the blocks showed the same or different identities.

**Fig. 3:**
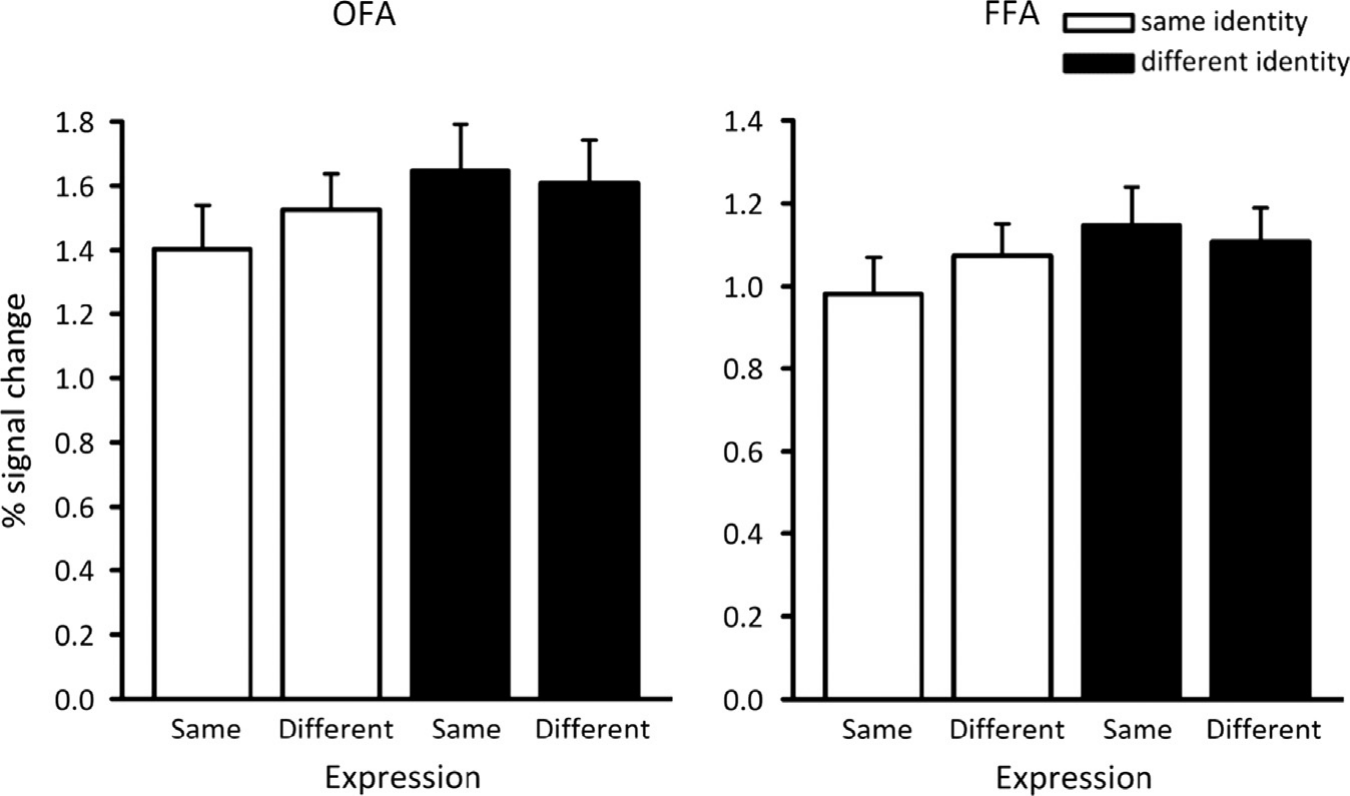
Peak responses to the different stimulus conditions in the OFA and FFA. Both regions showed a significant increase in response to changes in expression and to changes in identity.

The responses to the different conditions in the FFA are shown in Fig. 3. A 22×2 ANOVA revealed no significant main effect of Expression (*F*_(1,18)_=0.44) but there was a main effect of Identity (*F*_(1,18)_=6.37, *p*=0.02). There was a borderline but not significant Expression^⁎^Identity interaction (*F*_(1,18)_=3.48, *p*=0.08). The main effect of Identity was due to a bigger response to the *different-identity* conditions compared to the *same-identity* conditions. The OFA showed a similar pattern of response to that found in FFA. There was no significant effect of Expression (*F*_(1,18)_=0.73), but there was a significant effect of Identity (*F*_(1,18)_=10.15, *p*=0.01). There was also a significant Expression⁎Identity interaction (*F*_(1,18)_=4.47, *p*=0.05). The interaction was due to a significantly bigger response to *different-identity* condition compared to *same-identity* condition for the *same-expression change* (*t*_(18)_=3.31, *p*=0.004) but not for the *different-expression change* conditions (*t*_(18)_=1.59, *p*=0.39).

In summary, the results from this experiment reveal that the amygdala was sensitive to the emotion category, with a greater response to blocks of movies that varied in the category of emotion compared to blocks of movies displaying the same change in emotion. This is consistent with a more categorical representation of expression. This is dissociable form the response in the pSTS which did not discriminate between blocks with same change and different changes in expression.

## 4 Discussion

The aim of this experiment was to use an fMR-adaptation paradigm to explore the response to dynamic facial expressions of emotion across different face-selective regions. We found a dissociation between the neural representation of facial expression in the amygdala and other face-selective regions. The amygdala had a greater response to blocks of movies that morphed to different facial expressions of emotion compared to blocks of movies that always morphed to the same expression. Moreover, adaptation to dynamic changes in expression in the amygdala was invariant to changes in facial identity.

Models of facial expression perception have debated whether facial expressions are represented as belonging to discrete categories of emotion or as gradations along continuous dimensions (see Bruce & Young, 2012). Previously, we offered an alternative perspective to the longstanding controversy by showing that both categorical and continuous representations of expression are used by the brain (Harris et al., 2012). Pairs of faces were morphed to create equal physical changes between images that either fell within the same emotion category or crossed the boundary between different emotion categories. We showed that the amygdala was more sensitive to between than within-category changes (showing a more categorical representation of facial expression), whereas the pSTS was equally sensitive to within and between-category change (indicating a more continuous representation).

The results from the present study support the conclusion that the amygdala is involved in the categorical representation of facial expressions of emotion. Dynamic movies provide a novel and stringent test of categorical perception as inherent in all the movies are continuous changes in expression. A categorical representation of expression will therefore need to disregard these largely irrelevant continuous changes in expression in order to determine the emotion category. The amygdala demonstrated a neural pattern of response consistent with a region that is able to assign expressions to discrete categories of emotion. The more categorical representation of expression demonstrated in the amygdala is optimal for the proposed role of this region in processing biologically relevant signals pertinent to survival (Adolphs et al., 1999; Sander, Grafman, & Zalla, 2003). This is reflected in neuropsychological studies of patients with amygdala damage who have demonstrated impairments in emotion recognition (Adolphs et al., 1994; Anderson & Phelps, 2000; Young et al., 1995), which are often accompanied by an attenuated reaction to potential threats (Feinstein, Adolphs, Damasio, & Tranel, 2011; Sprengelmeyer et al., 1999).

Although we have demonstrated a more categorical response in the amygdala compared to the pSTS it does not necessarily imply that the amygdala is insensitive to changes in facial expression that do not result in a change to the perceived emotion. Indeed, both the category to which a facial expression belongs (its social meaning) and its intensity convey information pertinent to the observer. Consistent with this, a number of studies have shown that responses in the amygdala can be modulated by changes in the emotion′s intensity (Morris et al., 1996; Thielscher & Pessoa, 2007). Nevertheless, the key finding here is that using dynamic stimuli we provide support for our previous proposal for a dissociation between the way facial expressions of emotion are represented in the pSTS and amygdala.

A categorical response to facial expression may not necessarily be useful in all interactions and there are many everyday examples where a continuous representation of expression may be more useful. This is reflected in how the interpretation of facial expressions can be influenced by the context in which they are encountered (Russell & Fehr, 1987). Furthermore, even the basic emotional facial expressions can be quite variable in how they are displayed (Rozin et al., 1994). Together, these findings suggest a more flexible continuous representation is also required for judgements of facial expression. The sensitivity to all the changes in facial expression shown in the present study is not inconsistent with the idea that the pSTS could be the neural substrate for this continuous representation. This would fit with previous studies that have shown a continuous representation of facial expression in the pSTS (Harris et al., 2012; Said, Moore, Norman, Haxby, & Todorov, 2010). Together, these findings highlight the role of the pSTS in processing moment-to-moment signals important in social communication (Allison, Puce, & McCarthy, 2000).

Because of the considerable importance attached to the efficient processing of facial information, different neural subcomponents are thought to be optimally tuned to different facial signals. As such, models of face perception have proposed that invariant features of the face such as identity are processed largely independently of the more dynamic features (Haxby et al., 2000). In this study, we showed that the sensitivity of the response in the amygdala to changes in facial expression was largely independent of identity. In contrast, the OFA was sensitive to both changes in facial expression and facial identity. The FFA demonstrated a similar pattern of results to the OFA, showing sensitivity to identity and a borderline (although not statistically significant) sensitivity to changes in expression. The results in the OFA and FFA broadly reflect the pattern observed in the pSTS. These findings might therefore be seen as consistent with the notion that the FFA is involved in judgements of both identity and expression (Cohen Kadosh, Henson, Cohen Kadosh, Johnson, & Dick, 2010; Fox, Moon, Iaria, & Barton, 2009; Ganel, Valyear, Goshen-Gottstein, & Goodale, 2005). However, this possibility should be treated cautiously because the same pattern of results would be predicted in neural regions that are sensitive to any structural change in the face image. Indeed, recent studies have demonstrated that the FFA and OFA do not hold image invariant representations of faces (Davies-Thompson, Gouws, & Andrews, 2009; Pitcher, Walsh, & Duchaine, 2011; Xu, Yue, Lescroart, Biederman, & Kim, 2009).

In conclusion, using dynamic changes in facial expression we demonstrate a dissociation between the neural representation of expression in the pSTS and amygdala, with a more categorical representation of expression in the amygdala. The more categorical amygdala response is likely optimal for efficient processing of information pertinent to survival.

## Figures and Tables

**Table 1:** MNI coordinates (mm) of face-selective regions. Coordinates for the center of gravity were averaged across all participants. Standard error is reported in parenthesis.

Region	*n*	*x*	*y*	*z*
FFA	19			
L	18	−41 (*1.0*)	−54 (*1.5*)	−21 (*1.0*)
R	19	43 (*1.1*)	−55 (*3.2*)	−22 (*1.6*)
OFA	19			
L	15	−39 (*2.1*)	−84 (*1.5*)	−16 (*0.9*)
R	19	43 (*1.6*)	−80 (*2.0*)	−14 (*1.2*)
STS	18			
R		53 (*1.7*)	−51 (*2.6*)	4.7 (*1.0*)
Amygdala	16			
R		17	−9	−18
